# Couvelaire uterus resulting in haemoperitoneum

**DOI:** 10.1093/jscr/rjab618

**Published:** 2022-01-19

**Authors:** Yi Jia Lee, Katrina Calvert, Kedar Jape

## Abstract

First described by Dr Alexandre Couvelaire in 1911, a Couvelaire uterus is a rare complication of severe placental abruption (PA), which is diagnosed by the direct visualization of the uterus. There has been only one case report thus far that reported of an intraperitoneal bleed due to PA resulting in a poor outcome. We present a rare and serious case of Couvelaire uterus resulting in haemoperitoneum with a good outcome (live healthy infant, uterus preservation and total blood loss of 800 ml) in a primigravida with no prior risk factors and low-risk pregnancy other than a complete placenta praevia diagnosed during anatomy scan, which subsequently was reported as clear of cervix in third trimester ultrasound scan. She was diagnosed with placenta abruption and pre-eclampsia post-partum. Most cases of PA cannot be predicted or prevented. However, in some cases, close monitoring and timely decision-making can prevent adverse maternal and foetal outcomes.

## INTRODUCTION

First described by Dr Alexandre Couvelaire in 1911, a Couvelaire uterus is a rare complication (5% of all cases of abruption) of severe placental abruption (PA), which is diagnosed by the direct visualization of the uterus [1, 2]. A Couvelaire uterus manifests when a ruptured decidual spiral artery causing haemorrhage, bleeds into the decidua basalis and into the myometrium. As blood permeates into the uterine serous layer, it results in a blue-violet ecchymosis. In very rare instances, the bleed can extend into the serosa and into the peritoneal cavity causing a haemoperitoneum, which was first described in a case report by Bertholdt (2016) [3]. Its exact aetiology is still unknown and is usually associated with PA, which is a premature separation of placenta before delivery.

Classic clinical features of PA include abdominal pain, vaginal loss (blood or bloody amniotic fluid), tender uterus present between contractions and increased uterine tone, classically described as a ‘woody uterus’ [4].

We present a rare and serious case of Couvelaire uterus resulting in haemoperitoneum with a good outcome (live healthy infant and uterus preservation) in a primigravida with no prior risk factors and a low-risk pregnancy. She was diagnosed with PA and pre-eclampsia post-partum. Our case emphasizes that PA is a clinical diagnosis and that a high index of suspicion is required.

## CASE REPORT

A 31-year-old female primigravida presented with a sudden onset of generalized abdominal pain at 35 weeks and 3 days of gestation at 11 pm with no relief from simple analgesia. There was no vaginal bleeding. Normal foetal movements were reported. It was a low-risk pregnancy other than a prior diagnosis of complete placenta praevia (CPP) on anatomy ultrasound (US) which was clear of cervix at a subsequent 32-week scan. She had no significant past medical history.

On examination, there was generalized abdominal tenderness and an irritable uterus was noted. Her vital signs and cardiotocography (CTG) were normal. A bedside US revealed a single live intrauterine pregnancy, cephalic, with a posterior placenta with no notable retroperitoneal clot or separation and normal Dopplers. Urine dipstix revealed only trace blood. Her blood work revealed leucocytosis and neutrophilia. Her other blood work, including haemoglobin level, platelet count, C-reactive protein, liver, kidney function tests and coagulation profile, were unremarkable. Kleihauer-Betke was negative.

She was reassessed the next morning due to ongoing abdominal pain with no relief from analgesia. Her uterus was tender and palpated as ‘woody’ without any relaxation. CTG revealed a pseudosinusoidal trace. A decision for emergency caesarean section (CS) wasmade.

**Figure 1:**
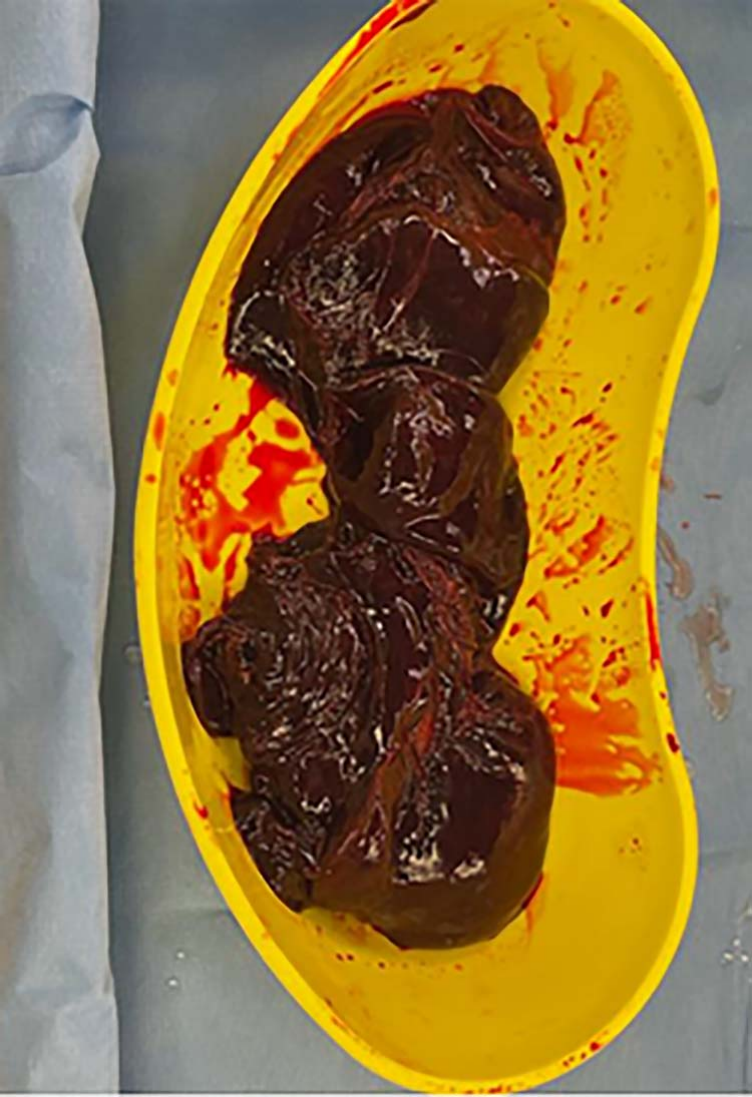
Retroperitoneal clot of 300 ml evacuated.

**Figure 2:**
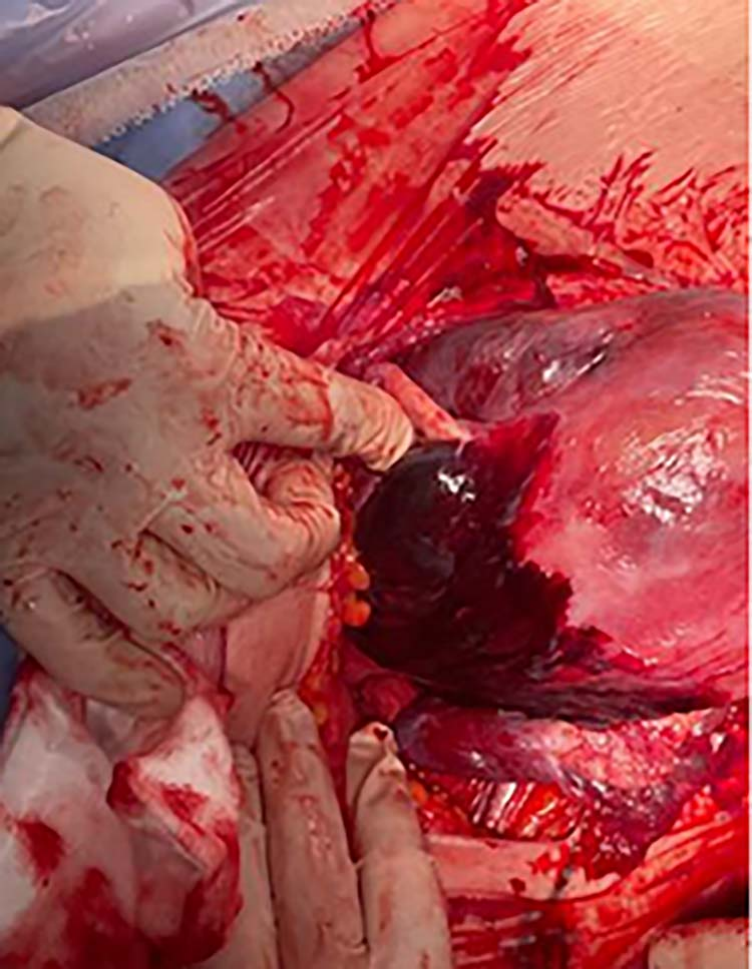
Couvelaire uterus.

**Figure 3:**
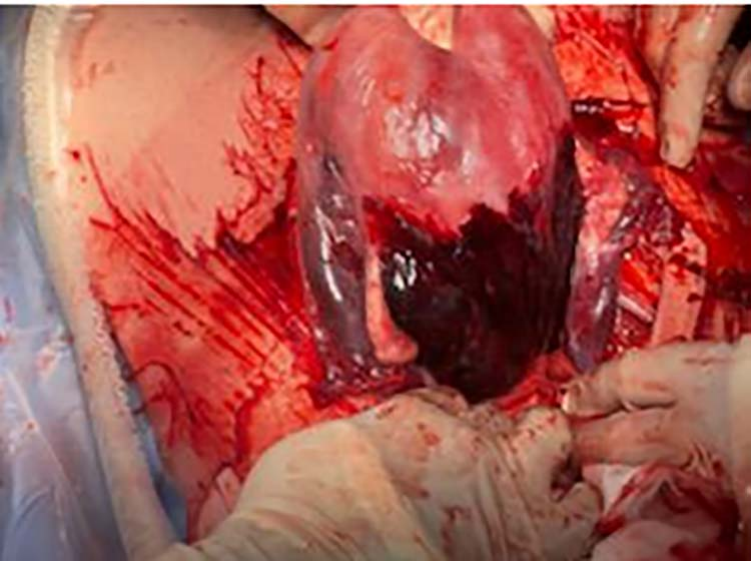
Bleeding from posterior wall of uterus.

On routine CS entry, there was a massive amount of blood at the level of rectus sheath. It was then immediately recognized that an intraperitoneal bleed was a high possibility. More blood was visualized on entry to the peritoneal cavity. On entry of the uterus, placenta was on view and a live male infant of 2940 g and the placenta was delivered quickly without difficulty. The uterus was exteriorized and 300 ml of retroperitoneal clot was evacuated (Fig. 1). There was active bleeding from the posterior uterine wall from the level of fundus to bilateral uterosacral attachment, with bleeding into the myometrium and serosal layers resulting in significant dark purple patch and prominent haematoma in the area (Figs 2 and 3).

This is diagnostic of a Couvelaire uterus with an associated intraperitoneal bleed. The uterus was repaired in two layers, and two interrupted haemostatic sutures plus fibrillar were applied to posterior serosa to achieve haemostasis. Poor uterine tone was noted and carboprost was given. Total blood loss was 800 ml. The newborn 1-minute and 5-minute Apgar scores were 6 and 10, respectively.

Her post-operative recovery was complicated by pre-eclampsia requiring magnesium sulphate infusion and regular anti-hypertensive medications.

## DISCUSSION

We present an unusual case of a Couvelaire uterus in which the haemorrhage has bled through the myometrium into the serosa with extensions intraperitoneally. The extent of the bleed signifies that the PA might have been developing for some time. The time between the onset of her abdominal pain to the time of delivery of the baby was about 14 hours.

To date, there is no set diagnostic clinical criterion for PA. According to the data from the New Jersey-Placental Abruption study [5], the most common indication leading to a clinical diagnosis of abruption was retroplacental clot(s) or bleeding (77.1%), followed by vaginal bleeding with uterine hypertonicity (27.8%) and vaginal bleeding with non-reassuring foetal status (16.1%).

Our patient initially presented with a vague clinical picture of generalized abdominal pain, normal CTG and a negative Kleihauer-Betke test. However, our patient at no point in time presented with or developed vaginal bleeding. It was only subsequently when she developed a tender uterus with increased tone and a non-reassuring CTG, there was a high clinical suspicion of concealed PA. Couvelaire uterus is more common in concealed haemorrhage.

It is crucial to have a high clinical suspicion of PA as affected patients do not always present with external vaginal bleeding in which PA can be concealed and that bedside US cannot always diagnose a fresh abruption. Hence, there is a risk of late or missed diagnosis leading to a high risk of intrauterine foetal death or hypoxic brain injury.

Another interesting point of note that might have contributed to the favourable outcomes for both mother and baby was the previous history of CPP diagnosed on the anatomy scan, which was reported to be clear in third trimester US. The placenta site being completely or partially within the lower uterine segment (LUS) may have assisted in keeping the blood loss low. The LUS has less muscle fibres, hence contractility, and may slow down the rate of placental separation from the myometrium leading to less serious symptoms which may distort the clinical picture. However, it may also lead to a favourable outcome.

The rapid progress of our case from mild initial symptoms to severe abruption with a Couvelaire uterus and features of intraperitoneal spread of the uterine haemorrhage, highlights the unpredictability of this clinical condition and the importance of close vigilance and timely decision-making, which can prevent adverse maternal and foetal outcomes.

## CONFLICT OF INTEREST STATEMENT

None declared.

## References

[1] Couvelaire A . Deux nouvelles observations d’apoplexie utero-placentaire (hemorrhagies retro-placentaires avec inltration sanguine de la pavoi musculaire del’uterus). Ann Gynecol Obstet 1912;9:486.

[2] Habek D, Selthofer R, Kulas T. Uteroplacental apoplexy (Couvelaire syndrome). Wien Klin Wochenschr 2008;120:88. 18322769 10.1007/s00508-008-0931-7

[3] Bertholdt C, Vincent-Rohfritsch A, Tsatsaris V, Goffinet F. Placental abruption revealed by hemoperitoneum: a case report. Am J Perinatol Rep 2016;06:e424–6. 10.1055/s-0036-1597267 PMC5161361 27994944

[4] Leeman L, Quinlan J, Dresang L, Gregory D. Advanced life support in obstetrics (ALSO) Provider Manual [Internet]. S3.amazonaws.com. 2017. http://s3.amazonaws.com/aafp/2017-also-provider/ALSO-Provider-2017-Syllabus.pdf (4 October 2021, date last accessed).

[5] Nath C, Smulian J, Shen-Schwarz S, Kaminsky L, Ananth C. Placental abruption is associated with histologic evidence of inflammation: the New Jersey-placental abruption study. Am J Obstet Gynecol 2006;195:S88. 10.1016/j.ajog.2007.06.012 17826437

